# A short note on hypertension management

**DOI:** 10.6026/97320630019307

**Published:** 2023-03-31

**Authors:** Shyamaladevi Babu, Rekha Kumari Dulala, Vadivel Mani, Sadhana Undru

**Affiliations:** 1 Faculty of Allied Health Sciences, Chettinad Hospital and Research Institute, Chettinad Academy of Research and Education, Kelambakkam-603103, Tamil Nadu, India; 2 Department of Biochemistry, Konaseema Institute of Medical Sciences and Research Foundation, Amalapuram, East Godavari district- 533201, Andhra Pradesh, India; 3 Department of Mental Health Nursing, Kim's College of Nursing, Konaseema Institute of Medical Sciences and Research Foundation, Amalapuram, East Godavari district-533201, Andhra Pradesh, India

**Keywords:** Hypertension, ethnicity, masked hypertension, lifestyle, stroke

## Abstract

Hypertension occupies a unique position in public health care, because it is the major cause of cardiovascular disease and the most frequent non-communicable disorder seen in primary care globally. The prevalence, treatment, and control rates of hypertension vary greatly by ethnicity. Such disparities are mostly linked to genetic variances, although lifestyle and socioeconomic level may influence health behaviours such as food - both of which appear to be substantial factors. East Asian communities have distinct ethnic traits. Hypertensive individuals are more likely to have salt sensitivity together with modest obesity. East Asians have a greater prevalence of stroke (especially hemorrhagic stroke) and no ischemic heart failure (HF) than Westerners. These problems are typical in both untreated and treated hypertensive patients. White coat hypertension affects 10%-30% of people who visit clinics for high blood pressure, whereas masked hypertension affects 10%-15% of people .With substantial hypertension populations in India and China, clinical studies in these areas are necessary to determine whether existing treatment techniques are effective. All patients with suspected or confirmed hypertension should receive regular lifestyle advice from their doctors, including recommendations for diet and exercise. Expert counsel and more frequent monitoring might be necessary.

## Background:

Hypertension occupies a unique position in public health care, because it is the major cause of cardiovascular disease and the most frequent non-communicable disorder seen in primary care globally. Raised blood pressure, in the absence of good prevention and management, dramatically increases the risk of stroke, myocardial infarction, chronic kidney disease, heart failure, dementia, renal failure, and blindness. There is an urgent need for stake holders across the health system, including individuals and families, researchers, and decision makers to collaborate to improve prevention, screening and detection, diagnosis and evaluation, awareness, treatment and medication adherence, management and control for people with or at risk of hypertension. Meeting this requirement will contribute to lowering the burden of hypertension-related illness, preventing complications and reducing the need for hospitalization and costly therapies [1].

Hypertension (HT) is one of the most frequent Non-Communicable Diseases and a serious public health problem, accounting for 19% of all NCD fatalities worldwide [2]. After air pollution and cigarette smoking, HT is projected to be the third largest cause of mortality and disability in South Asia [3]. Furthermore, it is an independent risk factor for coronary heart disease; HT's asymptomatic character leads to a lack of awareness of this ailment, earning it the nickname "silent killer disease." If left undetected or untreated, HT can greatly contribute to avoidable mortality and disability from coronary heart disease. As a result, it is critical to follow the core idea of levels of prevention in public health, which includes early diagnosis and timely treatment. India is the world's second most populated country, accounting for 18% of the global population [4], and it has one of the world's fastest expanding economies. In the SDG development indicators, India scored 131 out of 188 nations, with considerable variations across regions and states [5].

## Hypertension:

Hypertension in adults ( ) is defined as systolic blood pressure (SBP) of 140 mm of Hg or greater and / or greater and / or diastolic blood pressure (DBP) of 90mm of Hg or greater, based o the average of two or more properly measured, seated BP readings on each of two or more visits. According to most main standards, hypertension should be diagnosed when a person's systolic blood pressure (SBP) in the office or clinic is 140 mm Hg and/or their diastolic blood pressure (DBP) is 90 mm Hg after repeated testing. Table 1 shows a categorization of blood pressure based on office BP measurement, whereas Table 2O(see PDF) shows ambulatory and home BP readings used to define hypertension; both classifications apply to all people (over the age of 18). These blood pressure categories are intended to match treatment techniques to blood pressure levels [6].

High-normal blood pressure is meant to identify individuals who may benefit from lifestyle modifications and who may require pharmaceutical therapy if convincing reasons exist. Isolated systolic hypertension, characterized as high SBP (140 mm Hg) and low DBP (90 mm Hg), and is frequent in both children and the elderly. Isolated systolic hypertension is the most prevalent kind of essential hypertension in young people, including children, adolescents, and young adults. It is, however, more prevalent among the elderly, who have stiffness of the major arteries as well as an increase in pulse pressure. Individuals who have been diagnosed with verified hypertension (grades 1 and 2) should be given adequate pharmacological therapy [7].

## Ethnicity, Race and Hypertension:

The prevalence, treatment, and control rates of hypertension vary greatly by ethnicity. Such disparities are mostly linked to genetic variances, although lifestyle and socioeconomic level may influence health behaviours such as food - both of which appear to be substantial factors.

## Populations from Asia:

East Asian communities have distinct ethnic traits. Hypertensive individuals are more likely to have salt sensitivity together with modest obesity. East Asians have a greater prevalence of stroke (especially hemorrhagic stroke) and no ischemic heart failure (HF) than Westerners [8]. Morning and night time hypertension are also more frequent in Asian individuals than in European cultures. South Asian people from the Indian subcontinent are more vulnerable to cardiovascular and metabolic illnesses, such as CAD and type-2 diabetes. With substantial hypertension populations in India and China, clinical studies in these areas are necessary to determine whether existing treatment techniques are effective [9, 10,11].

## Risk Factors:

Unhealthy diets (excessive salt consumption, a diet heavy in saturated fat and Trans fats, a poor intake of fruits and vegetables), physical inactivity, cigarette and alcohol use, and being overweight or obese are all modifiable risk factors. A family history of hypertension, age over 65, and co-existing disorders such as diabetes or renal disease are non-modifiable risk factors [11].

## Symptoms:

Hypertension is referred to be a "silent killer". Most hypertensive persons are unaware of their condition since there are no warning signs or symptoms. As a result, it is critical that blood pressure be checked on a regular basis. Early morning headaches, nosebleeds, abnormal heart rhythms, visual alterations, and ear buzzing are some of the symptoms that might arise. Fatigue, nausea, vomiting, disorientation, anxiety, chest discomfort, and muscle tremors are all symptoms of severe hypertension. The only approach to identify hypertension is to have blood pressure measured by a medical expert. It is quick and painless to have your blood pressure checked. Individuals can test their own blood pressure using automated instruments, but an examination by a health expert is necessary for risk assessment and the identification of related diseases [12].

## White Coat and Masked Hypertension:

The usage of the workplace and out of the office (home or ambulatory) measurements identifies the Individuals with white coat hypertension who has high BP solely in the office (non-elevated ambulatory or home BP), whereas those with masked hypertension have no elevated BP in the office but elevated BP outside of the office (ambulatory or home). These problems are typical in both untreated and treated hypertensive patients. White coat hypertension affects 10%-30% of people who visit clinics for high blood pressure, whereas masked hypertension affects 10%-15% of people [13,14, 15].

## White coat hypertension:

These individuals have a cardiovascular risk that falls between that of normotensives and that of chronic hypertensives. Repeated office and out-of-office blood pressure readings are required to confirm the diagnosis. Drug therapy may not be suggested if their total cardiovascular risk is modest and there is no hypertension-mediated organ damage (HMOD). They should, however, be supplemented with lifestyle changes, since they may result in long-term hypertension that need pharmacological therapy [16, 17]

## Masked hypertension:

These individuals have the same risk of cardiovascular events as chronic hypertensives. The diagnosis must be confirmed with additional in-office and out-of-office measures. Masked hypertension may need medication therapy to regulate out-of-office blood pressure [18,19,20].

## Treatment:

All patients with suspected or confirmed hypertension should receive regular lifestyle advice from their doctors, including recommendations for diet and exercise. Even if a combined pill is used, dual therapy (for example, with an ACE and CCB) is not advised in the beginning because NICE found insufficient evidence regarding the risks and benefits of this approach and suggests further research is needed. At the very least, patients with hypertension should have an annual review to talk about their blood pressure, lifestyle, symptoms, and medication. If BP control with a single agent is insufficient, a second medication should be added. At the highest tolerated doses, step three treatment combines an ACE/ARB, CCB, and thiazide-like diuretic. Resistant hypertension is the term used to describe patients who continue to have high blood pressure despite this. Adherence should be examined, and ABPM or HBPM blood pressure readings should be validated. If additional therapy is required, patients with potassium levels below 4.5 mmol/l may benefit the most from low-dose spironolactone, while those with higher potassium levels may benefit more from an alpha- or beta-blocker. Expert counsel and more frequent monitoring might be necessary [21].

## Figures and Tables

**Table 1:** Hypertension classification based on office blood pressure (bp) measurement

**S:No**	**Category**	**Systolic (mmHg)**		**Diastolic (mmHg)**
1	Normal BP	<130	and	<85
2	High-normal BP	130–139	and/or	85–89
3	Grade 1 hypertension	140–159	and/or	90–99
4	Grade 2 hypertension	≥160	and/or	≥100

**Table 2:** Hypertension criteria based on office, ambulatory, and home blood pressure (hbpm) measurement

**S:No**		**SBP/DBP, mmHg**
1	Office BP	<130
ABPM		
2	24-h average	≥130 and/or ≥80
3	Day time (or awake) average	≥135 and/or ≥85
4	Night time (or asleep) average	≥120 and/or ≥70
5	HBPM	≥135 and/or ≥85
